# Guidance for the practical management of warfarin therapy in the treatment of venous thromboembolism

**DOI:** 10.1007/s11239-015-1319-y

**Published:** 2016-01-16

**Authors:** Daniel M. Witt, Nathan P. Clark, Scott Kaatz, Terri Schnurr, Jack E. Ansell

**Affiliations:** University of Utah College of Pharmacy, 30 South 2000 East, Salt Lake City, UT 84112 USA; Clinical Pharmacy Anticoagulation and Anemia Services, Kaiser Permanente Colorado, University of Colorado Skaggs School of Pharmacy and Pharmaceutical Services, Aurora, CO USA; Hurley Medical Center, Flint, MI USA; College of Human Medicine, Michigan State University, East Lansing, MI USA; Thromboembolism Research and Thoracic Surgery Research, St. Joseph’s Healthcare, Hamilton, ON Canada; Hofstra North Shore/LIJ School of Medicine, Hempstead, NY USA

**Keywords:** Anticoagulation, Warfarin, Coumadin, Venous thromboembolism, Anticoagulation clinics, Anticoagulation-related bleeding, Drug interactions, INR, Risk factors, Direct oral anticoagulants (DOAC)

## Abstract

Venous thromboembolism (VTE) is a serious and often fatal medical condition with an increasing incidence. The treatment of VTE is undergoing tremendous changes with the introduction of the new direct oral anticoagulants and clinicians need to understand new treatment paradigms. This article, initiated by the Anticoagulation Forum, provides clinical guidance based on existing guidelines and consensus expert opinion where guidelines are lacking. Well-managed warfarin therapy remains an important anticoagulant option and it is hoped that anticoagulation providers will find the guidance contained in this article increases their ability to achieve optimal outcomes for their patients with VTE Pivotal practical questions pertaining to this topic were developed by consensus of the authors and were derived from evidence-based consensus statements whenever possible. The medical literature was reviewed and summarized using guidance statements that reflect the consensus opinion(s) of all authors and the endorsement of the Anticoagulation Forum’s Board of Directors. In an effort to provide practical and implementable information about VTE and its treatment, guidance statements pertaining to choosing good candidates for warfarin therapy, warfarin initiation, optimizing warfarin control, invasive procedure management, excessive anticoagulation, subtherapeutic anticoagulation, drug interactions, switching between anticoagulants, and care transitions are provided.

## Introduction

Warfarin sodium remains an effective option for treating venous thromboembolism (VTE) despite a narrow therapeutic index, wide inter-patient dosing variability, predisposition to drug and food interactions, and need for close monitoring of the intensity of anticoagulation effect using the international normalized ratio (INR) [1]. This chapter will provide guidance for the optimal use of warfarin for VTE treatment.

## Background

Although clinical experience with warfarin spans over 6 decades, the evidence supporting consensus panel recommendations for many operational aspects of warfarin therapy is not strong [2]. As a result, warfarin therapy management is suboptimal in many cases [1]. This is important because failure to achieve adequate anticoagulation during VTE treatment predicts higher rates of subsequent recurrence [3–6]. Failing to achieve a therapeutic INR by day 5 of therapy also prolongs the inconvenience and pain associated with overlapping parenteral anticoagulation, and increases the length of hospitalization and overall treatment costs [2, 7]. Nonadherence to warfarin therapy during VTE treatment has also been associated with increased risk for recurrent VTE events [8].

## Methods

To provide guidance on the management of warfarin in patients with VTE, we first developed a number of pivotal practical questions pertaining to this topic (Table 1). Questions were developed by consensus of the authors. Guidance statements in this chapter were derived from evidence-based consensus statements whenever possible [2, 9–11]. The medical literature was reviewed for topics and key words including, but not limited to, coumarins, self care, point-of-care systems, administration and dosage, medication therapy management, drug monitoring, pharmacovigilance, sentinel surveillance, VTE, drug related side effects and adverse reactions, case management, patient care management, nomograms, algorithms, clinical decision support systems, pharmacists, nurses, physician assistants, and pharmaceutical services with a focus on high quality cohort studies and randomized controlled trials (RCTs) published since the most recent iteration of the American College of Chest Physician’s Evidence-Based Clinical Practice Guidelines on Antithrombotic Therapy and Prevention of Thrombosis (AT9). Guidance provided in this document is, whenever possible, based on the best available evidence. For some issues, however, published evidence is lacking, and in all instances, guidance statements represent the consensus opinion(s) of all authors and are endorsed by the Anticoagulation Forum’s Board of Directors.

**Table 1:** Guidance questions to be considered

Who are good candidates for warfarin therapy versus the direct oral anticoagulants?
How should warfarin be initiated?
How can I optimize anticoagulation control?
How do I manage warfarin during invasive procedures?
How do I manage warfarin-induced over-anticoagulation and bleeding?
How do I manage sub-therapeutic anticoagulation and recurrent VTE?
How do I manage warfarin drug–drug and drug-dietary interactions?
How do I switch between anticoagulants?
What is an appropriate follow-up and care transitions strategy?
How do I manage challenging clinical situations?

## Guidance

1. Who are good candidates for warfarin therapy vs the direct oral anticoagulants?

Based upon its pharmacokinetics, ability to be monitored, costs and other characteristics, warfarin may be the preferred anticoagulant for some patients and should be avoided in others.

*Patients with renal dysfunction*

Kidney disease is a risk factor for VTE [12]. In patients with estimated creatinine clearance (CrCl) between 30 and 50 mL/min there was no significant difference in the primary efficacy outcomes of recurrent thromboembolism or thromboembolism-related death with direct oral anticoagulants (DOACs) versus warfarin and the risk of major bleeding or the combined endpoint of major bleeding or clinically relevant non-major bleeding was also similar between treatments [13]. Little is known about the use of DOACs in patients with an estimated CrCl < 30 mL/min, as these patients were excluded from clinical trials comparing DOACs to warfarin for VTE treatment [14–20]. Because warfarin’s clearance does not rely on the renal route of elimination it is the preferred oral anticoagulant option for patients with an estimated CrCl < 30 mL/min [1]. However, decreased anticoagulation stability requiring more frequent and intensive management has been observed in patients with chronic kidney disease [21]. Unfractionated heparin is less dependent upon renal elimination than LMWH [22]. Therefore, UFH is preferred over LMWH during warfarin initiation for acute VTE in patients with severe renal impairment [9]. In general, RCTs comparing DOACs to warfarin employed the Cockroft–Gault equation to estimate renal function, used actual body weight, and did not round serum creatinine when estimating CrCl for the purposes of drug dosing and study exclusion criteria [23]. Since there is no consensus regarding which methodology most accurately predicts renal function for drug dosing, it is reasonable to employ a similar approach when selecting the most appropriate oral anticoagulant strategy for a given patient.

### **Guidance Statement**

*For patients with CrCl < 30 mL/min (estimated using the Cockroft–Gault equation) we suggest warfarin is the preferred anticoagulant. We also suggest vigilant monitoring including more frequent INR testing and bleeding risk assessment in patients with CrCl < 30 mL/min.*

*Patients with poor medication adherence*

Multiple daily dosing is known to decrease adherence [24]. Warfarin is administered once daily, as is edoxaban but apixaban and dabigatran are each administered twice daily [25–27]. Rivaroxaban is given twice daily for the first 21 days of acute VTE treatment, followed by once daily dosing thereafter [28]. Routine INR monitoring can identify poor medication adherence during warfarin therapy as out-of-range INRs often result from warfarin non-adherence [29]. In addition, repeated nonadherence to INR monitoring recommendations is easy to recognize and has been associated with increased thromboembolic risk and may therefore be a surrogate marker for non-adherence with taking warfarin doses as instructed [30]. Warfarin has a long half-life and isolated subtherapeutic INR excursions do not appear to substantially increase the risk of thromboembolism [31].

### **Guidance Statement**

*For patients with a history of poor medication adherence we suggest warfarin is the preferred oral anticoagulant. However, the requirement for routine INR monitoring of warfarin may be less than ideal for patients with restricted mobility, poor venous access, or other barriers to successful INR monitoring unless they are suitable candidates for self-testing at home using point-of-care INR monitoring devices (see below).*

*Patients with bleeding risk factors*

Compared to warfarin-based therapy (with heparin or LMWH overlap during initiation), apixaban resulted in a reduction in both major and clinically relevant non-major bleeding during treatment for acute VTE [20]. However, no difference in major bleeding was evident when dabigatran (although there was less overall bleeding) and edoxaban (showed less major plus clinically relevant non-major bleeding) when compared to warfarin-based VTE therapy [15, 18]. Interestingly, rivaroxaban was associated with less major bleeding when compared to warfarin-based therapy for treatment of PE, but not for DVT [16, 17]. Some of the bleeding associated with warfarin, dabigatran, and edoxaban may be attributable to the parenteral therapy used during initiation of therapy [15, 18].

It is unclear if DOACs should be used preferentially for patients with multiple bleeding risk factors because patients at high risk for bleeding were specifically excluded from the pivotal clinical trials. The use of DOACs in high bleeding risk patients is further complicated by a lack of specific reversal agent should bleeding occur [32]. There are several options to reverse warfarin in a bleeding patient; including vitamin K, fresh frozen plasma (FFP), and prothrombin complex concentrates (PCC) (see below) [1]. Appropriate intervention with these agents can rapidly normalize the INR in a bleeding warfarin patient. However, post hoc analyses of all major bleeding outcomes in phase III trials comparing warfarin and dabigatran did not indicate greater risk of morbidity or mortality with dabigatran-related bleeding compared to warfarin [33]. Nevertheless, lack of understanding of how best to manage of DOAC-related bleeding continues to challenge clinicians and presents a barrier to their use in the setting of a high bleeding risk patient in particular.

### **Guidance Statement**

*In patients at high risk for bleeding complications; warfarin has the advantage of a proven antidote and DOACs have less major and/or clinically relevant non-major bleeding. These factors need to be incorporated into shared decision making with patients.*

*Patients taking drugs known to interact*

Warfarin has many drug interactions, but dose adjustments based on INR monitoring can facilitate titration of the anticoagulant response following coadministration with interacting drugs [1]; this option is not available for DOACs. Drugs that inhibit or induce the P-glycoprotein efflux transporter result in significant alteration in serum concentrations of apixaban, dabigatran, edoxaban and rivaroxaban [32]. Rivaroxaban and apixaban are also metabolized by cytochrome P450 3A4 and influenced by inhibitors and inducers of this hepatic microsomal enzyme [32]. Package labeling provides some guidance regarding how common drug interactions may be managed for the DOACs. However, package labeling is limited to examples of drugs with known interaction potential and should not be considered a comprehensive list [34]. Therefore warfarin may be preferred for patients taking drugs known to interact with available DOACs or that have similar pharmacokinetic profiles.

Combining antiplatelet therapy with any type of anticoagulant increases bleeding risk [1, 35]. Compelling indications for concomitant antiplatelet therapy in patients taking warfarin for VTE are rare and poorly defined. Efforts should focus on limiting combined use to improve the safety of anticoagulant therapy wherever possible. Aspirin should not be added to anticoagulant therapy for primary prevention of coronary artery disease (CAD), including patients with diabetes (i.e. the “CAD equivalent”). In patients where combined anticoagulant-antiplatelet therapy is unavoidable, protection of the gastric mucosa with a proton pump inhibitor may be considered.

### **Guidance Statement**

*When avoiding drugs known to interact with a given anticoagulant is not an option, we suggest that warfarin is preferred because dose adjustments based on INR monitoring can facilitate titration of the anticoagulant response.*

*Patient preference and affordability*

Patient preference is an important consideration in selecting anticoagulation therapy for VTE treatment, and is influenced by factors related to convenience, comfort level, and the true out of pocket costs of a given anticoagulant. Clinicians should discuss with patients all costs of anticoagulation therapy, including copays, impact on drug coverage gaps and deductibles, charges for injectable anticoagulants during therapy initiation (if applicable), laboratory tests (including charges for tests performed at out-of-plan laboratories during travel), time away from work for laboratory visits, and transportation costs. For many patients, warfarin will remain the least expensive anticoagulant, even after non-medication costs are factored in. Patients initiating warfarin must also be willing and able to self-inject LMWH or fondaparinux during initiation of therapy if they are not hospitalized, or have a friend or family member assist with the injections. In addition, adherence to INR monitoring and dietary requirements is required. Cost-effectiveness analyses have investigated the financial impact of DOACs compared to conventional therapy for VTE treatment from the payer perspective. Rivaroxaban was cost-effective compared to enoxaparin and warfarin at a willingness to pay threshold of $50,000 per quality-adjusted life year [36]. Most of the cost savings in this analysis resulted from a shorter duration of stay during the index VTE hospitalization.

### **Guidance Statement**

*We suggest that anticoagulation providers thoroughly discuss the advantages and disadvantages of available anticoagulants with patients and initiate therapy for VTE based on appropriate selection criteria and patient preference.*

*Patients who are pregnant or breastfeeding*

Warfarin is a known teratogen and should not be used during pregnancy for management of VTE. Women of childbearing potential should be counseled to avoid becoming pregnant during warfarin therapy [1]. Warfarin therapy does not result in appreciable accumulation in breast milk and poses minimal risk to breastfeeding infants [37]. The DOACs are small molecules with potential to cross the placenta during pregnancy and since they have not been studied in human pregnancy they should be avoided in this setting [38, 39]. The mainstay for VTE treatment in pregnancy is LMWH although unfractionated heparin (UFH) has also been used successfully [37, 40].

### **Guidance Statement**

*For VTE treatment during pregnancy we suggest against using warfarin or DOACs. For VTE treatment in breastfeeding mothers we suggest that warfarin therapy is the best oral anticoagulant option.*

*Patients with antiphospholipid antibody syndrome*

Monitoring warfarin therapy using the INR can be challenging in some patients with antiphospholipid antibody (APLA) syndrome due to antibody interference with phospholipid-based coagulation assays [41]. Not all thromboplastin reagents are sensitive to these antibodies, so efforts should be made to select reagents less prone to APLA interference. Alternative tests, such as the chromogenic factor X assay, are also available for monitoring warfarin therapy in these special cases although test turn-around time may be several days. Little information is available regarding the use of DOACs, LMWHs, or fondaparinux in APLA syndrome and there have been case reports of treatment failure among patients transitioning between warfarin and DOACs for management of APLA syndrome [42].

### **Guidance Statement**

*For patients with VTE associated with APLA syndrome, we suggest warfarin adjusted to a target INR range 2.0–3.0 is the best option for long-term treatment* [43].

(2) How should warfarin be initiated?

Baseline laboratory measurements prior to warfarin therapy should include an INR for monitoring anticoagulant response, and a complete blood count with platelets. Warfarin should be initiated as soon as possible following diagnosis of VTE, preferably on the same day, in combination with UFH, LMWH or fondaparinux [9]. Individual responses to warfarin vary based on factors such as inpatient or outpatient status, age, genotype, concomitant medications, and comorbidities; however, the initial dose of warfarin should be 5 or 10 mg for most patients [1, 7]. Initial doses <5 mg might be appropriate in patients >75 years, the malnourished, those with liver disease or congestive heart failure, patients receiving medications known to inhibit warfarin’s metabolism, or patients with a high bleeding risk [1]. For patients sufficiently healthy to be treated as outpatients, warfarin 10 mg daily for the first 2 days has been suggested [2]. Initial warfarin doses >10 mg should be avoided [1]. Beginning on day three of therapy, INRs should be measured daily and warfarin doses adjusted to achieve an INR ≥ 2.0 as soon after day 5 of therapy as possible [9].

Daily INRs can be challenging for some patients due to geographic barriers and physical limitations. These barriers should be considered prior to anticoagulation initiation. In circumstances where daily INR monitoring is not possible, DOACs may be preferred. Dosing nomograms are available to assist with warfarin therapy initiation (Table 2); however, a recent meta-analysis comparing the efficacy of 10 and 5 mg warfarin nomograms among patients with VTE did not conclusively demonstrate the superiority of either approach for initiation of warfarin to achieve an INR of 2.0–3.0 on the fifth day of therapy [7].

**Table 2:** Example of a warfarin dose-initiation nomogram [107]

Day	INR	Warfarin dose (mg)
5-mg warfarin initiation nomogram		
1		5
2		5
3	<1.5	10
	1.5–1.9	5
	2.0–3.0	2.5
	>3.0	0
4	<1.5	10
	1.5–1.9	7.5
	2.0–3.0	5
	>3.0	0
5	<2.0	10
	2.0–3.0	5
	<3.0	0
6	<1.5	12.5
	1.5–1.9	10
	2.0–3.0	7.5
	>3.0	0

Small pilot studies evaluating the use of cytochrome 2C9 and vitamin K epoxide reductase (VKORC1) pharmacogenomic information to guide initiation of warfarin therapy demonstrated decreases in the time to reach a stabilized INR within the target therapeutic range, increases in time spent within the therapeutic range during early therapy, and decreases in the frequency of warfarin dose adjustments [44]. Subsequent large RCTs failed to confirm the clinical utility of pharmacogenomic testing [45–47]. The mean TTR during the first 12 weeks of warfarin therapy (including patients receiving treatment for VTE) was improved by a genotype-guided strategy in one RCT (adjusted difference, 7.0 percentage points; 95 % confidence interval, 3.3–10.6) and the median time to reach a therapeutic INR was 21 days in the genotype-guided group as compared with 29 days in the control group (p < 0.001) [46]. However, the fact that 21 days were required to achieve a therapeutic INR using the genotype-guided strategy should be troubling to clinicians striving to minimize the duration of overlapping parenteral therapy at the initiation of VTE treatment. The largest RCT to date (including 1015 patient, over half receiving treatment for VTE) found no differences in TTR (mean difference -0.2, 95 % CI −3.4 to 3.1) or a combined outcome of any INR ≥ 4.0, major bleeding or thromboembolism when a genotype-guided strategy was compared to one that used only clinical variables [47]. In a meta-analysis of 2812 patients enrolled in nine RCTs, a genotype-guided dosing strategy did not result in improved TTR (mean difference 0.14, 95 % CI −0.10 to 0.39), fewer patients with an INR greater than 4 (risk ratio 0.92, 95 % CI 0.82–1.05), or a reduction in major bleeding or thromboembolic events (risk ratios 0.60, 95 % CI, 0.29–1.22, and 0.97, 95 % CI, 0.46–2.05, respectively) compared with clinical dosing algorithms [45]. Furthermore, pharmacogenomic testing is not covered by many insurance plans including Medicare, is unlikely to be cost effective for general patients, and test results will likely not be available in time to affect initial warfarin dosing selection; therefore, pharmacogenomic testing to determine initial warfarin doses is not recommended for most patients [2, 48].

### **Guidance Statements**

*During warfarin initiation for VTE treatment we suggest the following*:

- *Initiate warfarin as soon as possible following diagnosis of VTE, preferably on the same day, in combination with UFH, LMWH or fondaparinux.*
- *The initial dose of warfarin should be 5 or 10 mg for most patients.*
- *Beginning on day 3 of therapy, INRs should be measured daily and warfarin doses adjusted to achieve an INR ≥ 2.0 as soon after day five of overlap therapy as possible.*
- *We suggest against using pharmacogenomic testing to determine initial warfarin doses for most patients.*

(3) How can I optimize anticoagulation control?

Well-managed warfarin therapy reduces the risk of adverse events including recurrent VTE and bleeding and the costs associated with VTE treatment [1]. Common factors linked to non-therapeutic INR results include changes in dietary vitamin K intake, concomitant medications, non-adherence, initiation phase of warfarin therapy, and changes in health status. Unfortunately, no reason can be identified for nearly half of non-therapeutic INRs [49]. Suggestions for optimizing warfarin therapy addressing these and other factors are discussed briefly below.

*Computer-aided warfarin dosing decision support*

A large observational study in Sweden showed that computer-aided warfarin dosing improved the probability of the next INR being in range in most cases compared to manual dosing [50]. In another RCT, INR outcomes associated with computer-aided warfarin dosing were non-inferior to those resulting from a simple paper-based dosing algorithm during maintenance warfarin therapy [51]. Computer-aided dosing of warfarin therapy may be most appropriate for high-volume centers, such as specialized anticoagulation clinics [2]. In a large RCT evaluating the impact of using computerized decision support on warfarin dosing provided by experienced anticoagulation providers, the subgroup of patients receiving treatment for VTE experienced improved therapeutic INR control and clinical outcomes [52].

### **Guidance Statement**

*When determining warfarin doses during VTE treatment we suggest using computer-aided warfarin dosing programs or validated dosing algorithms over an ad hoc approach.*

*Anticoagulation management services*

Inpatient and outpatient anticoagulant management services (AMS) evolved in order to address the challenges associated with managing and coordinating warfarin therapy [2]. Hallmarks of AMS care include sophisticated patient tracking systems, anticoagulation providers with specialized knowledge and skill focused predominantly on managing warfarin, comprehensive patient education, and outcome evaluation and quality improvement activities [53]. Well-coordinated follow up is especially critical during anticoagulation therapy initiation, when warfarin is being overlapped with parenteral anticoagulation [54]. While the evidence base supporting the superiority of AMS is not as strong as many would hope, a meta-analysis of available studies demonstrated improved clinical outcomes associated with AMS compared to usual care (Table 3) [55].

**Table 3:** Outcomes of anticoagulation management service versus usual care [55]

Outcomes	Events in AMS/patients (%)	Events in UC/patients (%)	Risk ratio (95 % CI)	I^2^ (%)
Major bleeding				
RCTs	5/367 (1)	10/368 (3)	0.64 (0.18–2.36)	12.2
Non-RCTs	49/4619 (1)	91/4595 (2)	0.49 (0.26–0.93)	46.7
Thromboembolic				
RCTs	8/367 (2)	11/368 (3)	0.79 (0.33–1.93)	0.0
Non-RCTs events	44/5335 (1)	133/5250 (3)	0.37 (0.26–0.53)	3.7
All cause mortality				
RCTs	10/299 (3)	11/299 (4)	0.93 (0.41–2.13)	0.0
Non-RCTs	5671/88,480 (6)	44,763/633,499 (7)	0.85 (0.37–1.98)	15.7

*AMS* anticoagulation management service, *UC* usual care, *CI*, confidence interval, *I* ^*2*^ measures the heterogeneity of pooled studies, *RCT* randomized controlled trial

For the individual physician without access to an AMS, the burden of anticoagulation management is significant. Education, tools, and tips are available on-line (e.g. The National Blood Clot Alliance (http://www.stoptheclot.org); The Anticoagulation Forum Centers of Excellence (http://excellence.acforum.org); and Clot Care (http://clotcare.com)). A standardized approach to warfarin dose initiation, familiarity with available guidelines, and leveraging support staff to facilitate tracking and/or a reminder system to monitor for patients who are overdue for INR lab work are critical elements to a successful anticoagulation management for individual prescribers [53]. An intervention consisting of prescriber education, clinical decision support, consultation triggers, and checklists helped to improve inpatient VTE management in one study even without an AMS [56].

### **Guidance Statement**

*We suggest enrolling patients with VTE in an AMS, but when such services are not available, individual clinicians should strive to implement a similar structured care process.*

*Patient self-testing and patient self-management*

Portable fingerstick INR devices enable patients to engage in self-testing and/or management at home [1, 2]. The accuracy of INRs measured using fingerstick devices is acceptable for clinical application when viewed within the context of the inherent limitations of the INR as a coagulation measure [57]. Patient self-testing (PST) involves patients performing their own INR at home and reporting their test results to a healthcare professional responsible for making warfarin-dosing decisions. Patient self-management (PSM) refers to properly trained, highly motivated patients independently altering their dose of warfarin therapy based on fingerstick INR results. In clinical trials PST and PSM are associated with higher levels of satisfaction with care, modestly improved therapeutic INR control (2.71 % TTR improvement, 95 % CI −6.1 to 11.51 % for patients with mechanical heart valves; 5.13 % improvement, 95 % CI 0.97–9.28 % for patients with atrial fibrillation), and lower risk for thromboembolic complications compared to those managed by ‘usual care’ (hazard ratio 0.51, 95 % CI 0.31–0.85), but RCTs evaluating PST/PSM outcomes in VTE-specific cohorts are lacking [2, 58]. In a subset of ‘real-world’ patients with DVT engaging in PST, those testing weekly had higher TTR (72.9 %) than those testing less frequently (65.8 %) [59]. PST has been used successfully in pediatric patients and may be associated with improved quality of life for these patients and their families [60–62]. Fingerstick INR devices are relatively expensive, but limited coverage of the monitor and testing strips is available for some patients, but not until after 90 days of treatment has been completed in some cases. The weekly INR monitoring generally used with PST/PSM has been associated with increased cost compared to traditional venipuncture monitoring [63].

### **Guidance Statement**

*For treatment of VTE, we suggest PST and PSM should be accompanied by patient education and reserved for motivated patients who can demonstrate competency with self-testing equipment.*

*Responding to slightly out-of-range INRs*

For patients with previously stable INR control, the single slightly out-of-range INR probably represents random variation; therefore adjusting warfarin doses may not be necessary and may in fact tend to destabilize the INR leading to suboptimal control [64]. For patients with previously stable therapeutic INRs presenting with a single out-of-range INR of ≤0.3 INR units below or above the therapeutic range, continuing the current dose and retesting the INR within 7–14 days may lessen the potential for destabilizing INR control [2, 65, 66]. Warfarin dose adjustments (increasing or decreasing the cumulative weekly warfarin dose by 5−20 %) should be made for persistently out of range INR values [50, 67]. The optimal process for adjusting the weekly warfarin dose has not been defined. Patients using a single strength of warfarin may need to administer different doses on some days of the week (e.g. one tablet Monday, Wednesday, and Friday and one half tablet on all other days). Patients using multiple warfarin strengths may be able to administer the same dose each day by combining tablet strengths. The method chosen should be individualized based on patient preference.

After a dose adjustment checking the INR during the next 2 weeks while a new warfarin steady state is being achieved can help determine whether further intervention is needed. Conservative dose adjustments can prevent overcorrection and dose destabilization and more frequent INR monitoring can ensure the return of therapeutic anticoagulation. Predicting when a new steady state is achieved after adjusting weekly warfarin doses is difficult and may be particularly problematic when patients require very low weekly doses and achieving steady state may take greater than two weeks. Evidence supporting skipping doses or administering one-time ‘boost’ doses to patients with slightly out-of-range INRs is limited and this common practice should be discouraged unless the out-of-range INR is associated with a temporary risk factor (e.g. missed dose, concurrent illness, presence of interacting drugs, dietary factors) [66].

### **Guidance Statement**

*For patients with previously stable therapeutic INRs presenting with a single out-of-range INR of ≤ 0.3 INR units below or above the therapeutic range we suggest continuing the current warfarin dose and retesting the INR within 7–14 days. We suggest against the routine use of boost or skipped warfarin doses for unexplained slightly out of range INRs*.

(4) How do I manage warfarin during invasive procedures?

Managing warfarin for invasive procedures requires an estimate of the risk of bleeding if warfarin is not interrupted compared with the risk of thromboembolism if warfarin therapy is interrupted [68]. A systematic review of primarily observational studies showed no decrease in thromboembolic events and an increase in bleeding associated with the use of LMWH bridging [69]. Results of the BRIDGE trial, a large randomized, controlled trial comparing to bridging with LMWH to placebo found that no bridging was noninferior to perioperative bridging with LMWH for the prevention of arterial thromboembolism and also decreased the risk of major bleeding [70]. This trial did not include patients with VTE. Observational data from patients with VTE interrupting warfarin for invasive procedures indicates that LMWH bridge therapy was associated with an increased risk of bleeding and is likely unnecessary for most of these patients as the risk for recurrent VTE was not different between patients who were and were not bridged. [71].

There is growing acceptance that the thromboembolic risk associated with interrupting warfarin outweighs the bleeding risk incurred by continuing warfarin during dental work, cataract surgery, minor dermatologic procedures and other procedures associated with minimal bleeding risk [68, 72]. However, for procedures with high bleeding potential, stopping warfarin 4–5 days prior to the procedure is necessary to return the INR to near-normal values (Table 4). The risk of recurrent VTE is low, even when near-normal INR values are required prior to the procedure; therefore most patients with VTE can safely interrupt warfarin for invasive procedures without bridge therapy [71]. Criteria for determining which patients are at recurrent VTE risk high enough to require bridge therapy are not well defined, but bridge therapy using LMWH has been consistently associated with at least a three-fold increased risk for major bleeding [69–71, 73]. Therefore, consideration of bridge therapy should be reserved for those at highest recurrent VTE risk (e.g. VTE within the previous month, prior history of recurrent VTE during anticoagulation therapy interruption, undergoing a procedure with high inherent risk for VTE such as joint replacement surgery or major abdominal cancer resection) [71, 74]. Even in these high-risk patients, the risk of bleeding associated with LMWH bridging must be carefully considered [74]. The use of prophylactic rather than therapeutic dose LMWH may also be considered.

**Table 4:** Suggested approach to warfarin therapy interruption for invasive procedures

Days from Procedure	Anticoagulation management
7–14 days before	Assess recurrent VTE and procedure-related bleeding risk
	If high risk for VTE recurrence consider bridging with LMWH (unnecessary for most patients with VTE)
	Obtain baseline INR and determine number of warfarin doses to hold prior to procedure
7 days before	Stop aspirin or other antiplatelet therapy if deemed safe and necessary
4 or 5 days before	Stop warfarin
2 or 3 days before	Start LMWH if necessary^a^
Day before	Give last dose of LMWH 24 h before procedure^b^
	Verify INR is low enough to proceed with procedure Give vitamin K 2.5 mg orally if INR above goal for procedure
Day of	Resume usual maintenance warfarin dose^c^ after procedure
1–3 days after	Resume LMWH if necessary^d^
	Resume aspirin or other antiplatelet therapy once adequate hemostasis is verified
5 + days after	Stop LMWH once INR is therapeutic

^a^LMWH usually initiated approximately 72 h prior to the procedure

^b^Give only the morning dose of twice-daily therapeutic-dose LMWH and reduce once-daily therapeutic-doses by 50 %

^c^Using “booster” doses (e.g. 1.5–2 times the usual dose) for 1–2 days when resuming warfarin therapy may reduce time required to achieve INR ≥ 2.0 [108]

^d^Resume LMWH approximately 24 h after (e.g. the day after) the procedure for lower bleeding risk procedures; for high bleeding risk procedures wait 48 to 72 h and ensure adequate hemostasis before resuming LMWH, or avoid LMWH completely [68, 109]

*INR* international normalized ratio, *LMWH* low-molecular-weight heparin

### **Guidance Statements**

*For patients requiring invasive procedures during warfarin therapy for VTE we suggest the following*:

- *Careful coordination of periprocedural warfarin management involving the anticoagulation provider, person performing the procedure, and the patient*.
- *Continuing warfarin during dental work, cataract surgery, minor dermatologic procedures and other procedures associated with minimal bleeding risk over interrupting warfarin therapy*.
- *Stopping warfarin 4–5 days prior to the procedure to return the INR to near-normal values for procedures with high bleeding potential*.
- *No bridge therapy for most patients (exceptions might include VTE within the previous month, prior history of recurrent VTE during anticoagulation therapy interruption, undergoing a procedure with high inherent risk for VTE such as joint replacement surgery or major abdominal cancer resection)*.

(5) How do I manage warfarin-induced over-anticoagulation and/or bleeding?

Most patients with asymptomatic INR elevations should be managed simply by withholding warfarin therapy until the INR has decreased to a safer level nearer the therapeutic range (see Question #3) [2]. Reversing anticoagulation with vitamin K has not been shown to affect the risk of major bleeding for asymptomatic INRs between 4.5 and 10 compared to withholding warfarin alone [75]. However, withholding warfarin therapy alone may not lower high INRs quickly enough in patients at high risk for bleeding or when the return to a safer INR is expected to be delayed (e.g. INR elevation due to interacting medications, in patients taking low weekly warfarin doses, or those with history of prolonged INR elevation) [64]. Vitamin K 2.5 mg administered orally is suggested for non-bleeding patients with INRs > 10 [76]. Orally administered vitamin K is preferred over the intravenous route in the absence of major bleeding [2]. Subcutaneously administered vitamin K should be reserved as a last resort due to unpredictable absorption [77].

Management of warfarin-associated major bleeding should include general supportive care and bleeding site interventions along with rapid reversal of anticoagulation [2, 78]. Restoration of hemostasis with vitamin K requires synthesis of new clotting factors by the liver, a process requiring at least 6 h and more likely 12–24 h to have a significant impact on lowering the INR. Infusion of FFP replenishes functional clotting proteins more rapidly, but requires blood-type cross matching, thawing prior to administration, and large volumes in many cases which can result in fluid overload especially in patients with heart failure or renal dysfunction [79]. The estimated volume of FFP needed to significantly reduce the INR, depending on its degree of elevation, is between 15 and 30 mL/kg. PCC contain concentrated lyophilized clotting proteins that do not require thawing or blood-type cross matching, and can be rapidly administered after reconstitution without risk of fluid overload and with a negligible risk of viral transmission and transfusion-related acute lung injury [79]. Three- and 4-factor PCC both contain factors II, IX, X, and proteins C and S, but 3-factor PCCs contain little or no factor VII while 4-factor PCC contains significant amounts of factor VII. Four-factor PCC was shown to be non-inferior to FFP for the outcome of hemostatic efficacy 24-h following administration [80]. However, 4-factor PCC is preferred over FFP due to more rapid reversal of anticoagulant effect, ease of preparation and administration, and less potential for volume overload [81]. Three-factor PCC (with or without supplemental FFP as a source of factor VIIa) can be used instead of 4-factor PCC if necessary. Activated PCC, which contains factor VIIa in addition to factors II, IX and X, may be associated with a theoretically higher risk of thromboembolic events than non-activated products [79]. Recombinant factor VIIa is not recommended for warfarin reversal because it only replaces 1 of the 4 clotting factors inhibited by warfarin and its high cost [79]. Because the half-life of factor VIIa is only 6–8 h compared to warfarin’s half-life of 36 h, both PCC and FFP should be administered in combination with 5–10 mg of vitamin K via slow IV injection to ensure sustained warfarin reversal [2].

### **Guidance Statements**

*For non-bleeding patients presenting with an elevated INR we suggest the following*:

- *Withholding warfarin alone or in combination with 1.25–2.5 mg of oral vitamin K for INRs between 4.5 and 10.0*.
- *2.5 mg of oral vitamin for INRs > 10.0.*

For warfarin-related major bleeding we suggest rapid reversal of anticoagulation with 5–10 mg intravenous vitamin K and 4-factor non-activated PCC in conjunction with general supportive care and bleeding site interventions.

(6) How do I manage sub-therapeutic anticoagulation and recurrent VTE?

Outside of the initial 30 days of VTE treatment, the risk of thromboembolic events in patients with stable INRs experiencing a single sub-therapeutic INR value is not sufficiently high to warrant bridge therapy with injectable anticoagulants [31]. Management strategies for recurrent VTE during ongoing anticoagulation depend on factors such as medication adherence, INR control in the period preceding the recurrent event, presence of malignancy, and proximity to the initial VTE event. Determining and correcting the causes of non-adherence to anticoagulation therapy should occur for VTE recurrence attributable to medication non-adherence. This may involve switching to an alternative anticoagulant depending on individual circumstances. It may not be necessary to increase the targeted intensity of anticoagulation therapy when a period of sub-therapeutic anticoagulation precedes recurrent VTE. Conversely, increasing the targeted anticoagulation intensity may be required for patients who experience recurrent VTE despite therapeutic anticoagulation [82]. Patients with cancer experiencing recurrent VTE during warfarin therapy should be switched to LMWH [9, 83].

### **Guidance Statement**

*For most patients with VTE and subtherapeutic warfarin anticoagulation we suggest re-establishing therapeutic anticoagulation as quickly as possible without bridge therapy. For recurrent VTE not associated with subtherapeutic warfarin anticoagulation we suggest either increasing the target INR range or switching to an alternative anticoagulant*.

(7) How do I manage warfarin drug–drug and drug-dietary interactions?

Pharmacokinetic drug interactions with warfarin are primarily related to drugs that inhibit or induce hepatic metabolism via the cytochrome 2C9, 1A2, and 3A4 isoenzymes [1]. Pharmacodynamic interactions increase the bleeding risk associated with warfarin by affecting hemostasis, platelet function, or clotting factor catabolism [84]. When drugs with known interaction potential are added or discontinued during warfarin therapy, or there is uncertainty regarding a drug’s potential to alter the response to warfarin, more frequent INR testing should be performed until INR stability is re-established [1, 84]. Incorporating drug interaction alerts into electronic health records has been shown to improve the rate of subsequent INR monitoring [85]. Acute infections or other changes in health status (e.g. fever, heart failure, diarrhea, vomiting) can alter warfarin response regardless of whether antibiotics are prescribed and should also prompt more frequent INR monitoring [86].

Many foods contain sufficient vitamin K to reduce the anticoagulation effect of warfarin if consumed repetitively or in large portions [1]. Consistency in dietary vitamin K intake should be stressed rather than abstinence, as high daily intake of vitamin K has been associated with more stable INR control [87]. Significant changes in dietary vitamin K intake should prompt more frequent INR monitoring similar to when interacting medication are co-administered.

### **Guidance Statement**

*Following co-administration of drugs with the potential to interact with warfarin or significant changes in dietary vitamin K intake we suggest more frequent INR testing and warfarin dose titration as needed until INR stability is re-established*.

*Vitamin K supplementation*

Changes in dietary vitamin K intake may influence INR stability during warfarin therapy and several studies evaluating the impact of reducing fluctuations in dietary vitamin K intake via daily low-dose vitamin K supplementation, have been published [88–91]. These studies do not support daily supplementation with low doses of vitamin K as a means to improve therapeutic INR control or clinical outcomes during warfarin therapy [2, 90, 92].

### **Guidance Statement**

*We suggest against routine use of daily low-dose vitamin K supplements as a means to improve therapeutic INR control or clinical outcomes during warfarin therapy*.

(8) How do I switch between anticoagulants?

Transitions from warfarin to other anticoagulants are described in the chapters providing guidance for parenteral anticoagulants and DOACs and in Tables 5 and 6. Transitioning from other anticoagulants to warfarin is detailed below.

**Table 5:** Switching to DOACs

Warfarin to DOAC	
Dabigatran^a^	Start when INR < 2.0
Rivaroxaban^a^	Start when INR < 3.0
Apixaban^a^	Start when INR < 2.0
Edoxaban^a^	Start when INR ≤ 2.5
LMWH to DOAC	
Dabigatran	Start DOAC within 0–2 h of the time of next scheduled dose of LMWH
Rivaroxaban	
Apixaban	
Edoxaban	
(iv) UFH to DOAC	
Dabigatran^a^	Start DOAC immediately after stopping iv UFH
Rivaroxaban^a^	
Apixaban^a^	
Edoxaban^a^	Start Edoxaban 4 h after stopping iv UFH

As a general rule, we suggest that as INR drops below 2.5, a DOAC can be started

As a general rule, we suggest that each DOAC can be started within 30 min after stopping (iv) UFH

^a^Recommendations adapted from company’s package insert

**Table 6:** Switching to warfarin

DOAC to warfarin	
Dabigatran^a^	Start warfarin & overlap with dabigatran; CrCl ≥ 50 mL/min, overlap 3 days CrCl 30-50 mL/min, overlap 2 days CrCl 15-30 mL/min, overlap 1 day
Rivaroxaban^a^ Apixaban^a^	Stop DOAC; start warfarin & LMWH at time of next scheduled DOAC dose and bridge until INR ≥ 2.0
Edoxaban^a^	For 60 mg dose reduce dose to 30 mg & start warfarin concomitantly. For 30 mg dose reduce dose to 15 mg and start warfarin concomitantly. Stop edoxaban when INR ≥ 2.0

As a general rule, we believe either approach (i.e. stop DOAC then start LMWH & warfarin; or overlap warfarin with DOAC, measure INR just before next DOAC dose and stop DOAC when INR ≥ 2.0) can be used for all DOAC to warfarin transitions

*CrCl* creatinine clearance

^a^Recommendations adapted from company’s package insert. Overlap intended to avoid under-anticoagulation while warfarin effect developing. When DOAC overlapped with warfarin, measure INR just before next DOAC dose since DOAC can influence INR

*Dabigatran*

According to product labeling patients with a CrCl ≥ 50 mL/min transitioning from dabigatran to warfarin should initiate warfarin therapy and overlap with dabigatran for three days at which point dabigatran should be discontinued and warfarin doses adjusted to achieve an INR ≥ 2.0 as quickly as possible [25]. For patients with a CrCl between 30 and 50 mL/min and 15 and 30 mL/min, warfarin and dabigatran should be overlapped for two days and one day, respectively [25]. However, achieving a therapeutic antithrombotic effect takes much longer prompting some experts to suggest at least 3 days of overlap combined with measuring the INR just before the next dose of dabigatran to limit any effect on the INR. Using this method, dabigatran is stopped when the INR is 2.0 or higher. The safety and efficacy of this method compared to the instructions provided in product labeling is unknown.

*Rivaroxaban, apixaban, and edoxaban*

Rivaroxaban and, to a lesser extent apixaban and edoxaban, prolong the INR making INR measurements unreliable while transitioning to warfarin [26, 28, 93]. There are no clinical trials to direct how best to switch patients from these medications to warfarin. Product labeling suggests stopping rivaroxaban, apixaban, or edoxaban and starting warfarin in conjunction with a therapeutic dose of parenteral anticoagulant when the next DOAC dose is due and overlapping therapy for at least five days and the INR is ≥ 2.0 [26, 28, 93]. This approach may be reasonable in patients at high risk of VTE recurrence, but is probably unnecessary for most patients with VTE. Alternatively, warfarin and DOAC could be overlapped for 3 days followed by measuring the INR just prior to the dose of DOAC with the assumption of some DOAC influence on the INR (rivaroxaban > apixaban, edoxaban). The DOAC can be discontinued once the INR is ≥2.0. The product labeling for edoxaban suggests reducing the usual edoxaban by half at the start of overlapping therapy and measuring INRs at least weekly [93].

### **Guidance Statements**

*If a patient with VTE requires a switch from a DOAC to warfarin, we suggest one of the following approaches*:

- *Follow the information contained in the applicable product labeling.*
- *For patients at low risk of VTE recurrence, we suggest avoiding LMWH bridge therapy.*
- *For patients at high risk of VTE recurrence, we suggest overlapping apixaban, rivaroxaban, edoxaban, or dabigatran with warfarin therapy for at least 3 days combined with measuring the INR just before the next DOAC dose to limit any DOAC effect on the INR and discontinue DOAC therapy when an INR ≥ 2.0 is achieved.*

(9) What is an appropriate follow-up and care transitions strategy?

The appropriate length of time between INR tests (INR recall interval) depends on prior INR stability and the probability of events in the foreseeable future that might affect the INR. When warfarin dose adjustments are necessary, a cycle of more-frequent INR monitoring should be completed until a consistent pattern of stable therapeutic INRs can be re-established [2]. An analysis in the Veterans Administration health care system suggested that the optimal recall interval after INRs ≥ 4.0 or ≤1.5 is within 7 days, and within 14 days following INRs 3.1–3.9 or 1.6–1.9 [94]. Choosing an INR recall interval that allows sufficient time for dosing changes to be reflected by the next INR will reduce the likelihood of introducing unwanted variation in the INR response. The INR recall interval should not exceed 3 days following an INR > 5.0 and if vitamin K is administered, the INR should be rechecked the next day to minimize the risk of INR overcorrection [95]. During acute VTE treatment when warfarin is being overlapped with parenteral anticoagulation therapy, INRs should be checked daily beginning on day three of therapy for at least three more days and the INR is ≥2.0 [9]. During the first 3 months of warfarin therapy for VTE, INR recall intervals should not exceed 6 weeks. After 3 months, INR recall intervals of up to 12 weeks are reasonable for patients demonstrating consistently stable INRs [2].

It is very common for patients with VTE to transition between healthcare settings at some point during anticoagulation therapy. In patients admitted to the hospital for initial treatment of VTE, the use of a pharmacist directed inpatient anticoagulation service has shown improvements in transitions of care communication with outpatient physicians and anticoagulation staff and timeliness of INR monitoring [96]. As many as 12 % of the more than 1.6 million Americans currently residing in nursing homes are receiving long-term anticoagulant therapy with warfarin and suboptimal communication between nursing home staff and prescribing physicians has resulted in patient safety issues [97]. Conversely, coordination of warfarin therapy for nursing home residents by dedicated anticoagulation providers has been shown to improve INR control [98]. Facilitated telephone communication between nurses and physicians using a structured approach has also been shown to modestly improve the quality of warfarin management for nursing home residents [97]. All patients and their caregivers should receive patient-centered education regarding warfarin use for VTE treatment (Table 7). Particular attention should be focused on recognition of high-risk situations that could compromise patient safety (e.g. recurrent VTE symptoms, stroke symptoms after incidental head trauma, symptoms indicative of internal bleeding) [99]. Patients completing overlapping therapy with warfarin and parenteral anticoagulation at home should have access to a provider 24 h a day, 7 days a week to facilitate addressing questions and concerns in a timely manner [54]. Anticoagulation management services have been shown to be beneficial in this regard [100].

**Table 7:** Warfarin patient education for venous thromboembolism therapy

General information regarding VTE and treatment goals
Anticoagulant medications prevent blood clots from growing larger while the body begins to dissolve the clot
The clot may completely dissolve with time, or may never go completely away; some people will have chronic pain and swelling in the affected limb; people with one clot are at increased risk of future clots
Warfarin tablets take several days to begin working; LMWH or fondaparinux injections work right away and provide protection against future clotting until warfarin is fully active
Warfarin tablets are taken for 3 months or longer to prevent blood clots from returning
It is important to take warfarin exactly as directed
Blood test monitoring
Regular blood tests called the international normalized ratio (INR) are required to make sure warfarin is working properly
The INR tells how quickly blood clots form
The goal INR range is between 2.0 and 3.0; risk for clotting is higher when INRs are less than 2.0, risk for bleeding is higher when INRs are greater than 3.0; doses of warfarin are adjusted based on INR test results
Other blood tests may be needed during warfarin therapy to help detect internal bleeding
Warfarin information
Each strength of warfarin has a unique color; with each warfarin refill make sure new tablets are the same color; if not, ask the pharmacist why
Warfarin should be taken at approximately the same time each day, preferably in the evening or at bedtime
Bleeding is the most common and serious side effect of warfarin; be careful to avoid injury
Warfarin has many drug interactions; always check with an anticoagulation provider before taking any new medications (including over-the-counter medications and dietary supplements)
Foods with a lot of vitamin K like broccoli, spinach, and green tea may interfere with warfarin; do not avoid foods with vitamin K, but try to maintain consistent dietary habits
Alcohol increases the risk for bleeding and interferes with warfarin therapy; no more than 1–2 drinks per day, and avoid binge drinking
Contact an anticoagulation provider if any of the following happen:
Bleeding from a cut or scrape that won’t stop
Blood in urine
Blood in stool
Nose bleeding that won’t stop
Increased swelling or pain in the area where the blood clot formed
Go to the emergency department if any of the following happen:
Shortness of breath
Chest pain
Coughing up blood
Vomiting up blood or material that resembles coffee grounds
Black tarry-appearing stool
Severe headache of sudden onset
Slurred speech

*DVT* deep vein thrombosis, *INR* international normalized ratio, *LMWH* low-molecular-weight heparin

### **Guidance Statements**

- *For patients requiring warfarin dose adjustments for out of range INRs we suggest rechecking the INR within 7 days after INRs ≥ 4.0 or ≤1.5, and within 14 days following INRs 3.1–3.9 or 1.6–1.9.*
- *Following an INR > 5.0 we suggest rechecking the INR within 3 days.*
- *Following vitamin K administration for excessive anticoagulation we suggest rechecking the INR the next day.*
- *When warfarin is being overlapped with parenteral anticoagulation therapy during initiation of acute VTE treatment we suggest that INRs be checked daily beginning on day 3 of therapy until the INR is ≥2.0.*
- *During the first 3 months of warfarin therapy for VTE we suggest that INR recall intervals not exceed 6 weeks.*
- *For patients demonstrating consistently stable INRs after 3 months of warfarin therapy for VTE we suggest that INR recall intervals can be extended up to 12 weeks.*
- *When patients receiving warfarin for VTE therapy transition between healthcare sites we suggest dedicated anticoagulation providers assume responsibility for care coordination using a structured approach*.
- *We suggest that all patients and their caregivers receive patient-centered education regarding warfarin use for VTE treatment at the initiation of therapy and periodically thereafter.*

(10) How do I manage challenging clinical situations?

*Liver disease*

Patients with liver disease receiving warfarin have poorer anticoagulation control and more bleeding [101]. A 4-point score system has been proposed to identify patients with liver failure at higher risk for poor anticoagulation control and bleeding during warfarin therapy (one point each for albumin [2.5–3.49 g/dL] or creatinine [1.01–1.99 mg/dL], and 2 points each for albumin [<2.5 g/dL] or creatinine [≥2 mg/dL]. Compared to patients without liver disease, those with liver disease and a score of zero had modestly lower TTR (56.7 %) but no increase in bleeding (hazard ratio, 1.16; p = 0.59), whereas those with liver disease and the worst score (4) had both poor control (29.4 %) and high risk of bleeding (hazard ratio 8.53; p < 0.001) [100]. Warfarin should not be used in patients with liver disease who have multiple or serious medical comorbidities (e.g. advanced pulmonary/heart disease, advanced stage hepatocellular carcinoma, recent history of GI bleeding), history of multiple falls, and inability to closely monitor anticoagulation [102]. In patients where the risk/benefit is deemed acceptable, warfarin should be initiated with a dose of 1 mg daily and carefully titrated to achieve an INR between 2 and 3. Esophageal varices should be banded prior to initiating warfarin therapy to reduce the risk of bleeding [102]. Portal vein thrombosis, a frequent complication in patients with end stage liver disease, can be safely managed through the careful use of warfarin therapy [102].

### **Guidance Statements**

- *For patients with liver disease complicated by VTE we suggest against warfarin in patients with multiple or serious medical comorbidities and inability to closely monitor anticoagulation.*
- *When warfarin therapy is deemed necessary in patients with liver disease we suggest a starting dose of 1 mg daily with careful dose titration to achieve an INR between 2 and 3.*
- *For patients with esophageal varices who require warfarin therapy we suggest banding of varices prior to beginning therapy when possible.*

*Nonadherence*

Repeatedly missing INR tests has been associated with an increased risk for thromboembolic complications during warfarin therapy [30]. A systematic process for tracking patients (e.g. an electronic database or tickler filing system) should be used to minimize the possibility that a patient on warfarin therapy is lost to follow-up, even for a brief period [53]. Patients who miss INR tests should receive non-threatening reminders via phone, text message, email, or regular mail. Patients at higher risk for bleeding or recurrent VTE should receive INR reminders as soon as possible. The reasons underlying nonadherence to INR monitoring instructions should be investigated and remedied when possible. Careful consideration of medical-legal implications should take place before patients who repeatedly fail to adhere to INR monitoring instructions are discharged from anticoagulation monitoring services and returned to the care of the referring provider. Patients who fail to follow warfarin dose instructions have a lower TTR on average and are likely at increased risk for thrombosis and potentially bleeding [29]. Pillboxes, calendars, diaries, electronic reminders, and written instructions can help patients remember to take their medications as prescribed [103].

### **Guidance Statements**

- *To ensure patients on warfarin are not lost to follow up we suggest use of a systematic tracking process.*
- *For patients who miss scheduled INR tests we suggest the use of non-threatening reminders delivered via phone, text message, email, or regular mail.*
- *For patients having difficulty following warfarin dose instructions we suggest anticoagulation providers explore the use of pillboxes, calendars, diaries, electronic reminders, and written instructions as means to improve adherence.*

*Travel*

Patients traveling during warfarin therapy should ensure a sufficient supply of warfarin is available to cover the duration of travel, and make arrangements for ongoing INR monitoring if necessary [104]. Patients should clearly understand their responsibility with regard to payment for INR testing at laboratories outside of their health plan service area. Patients should be aware of the implications of changes in normal dietary habits and alcohol consumption during travel, and what to do if symptoms of bleeding or recurrent VTE should occur. The potential effect of antibiotics prescribed for prevention of travel-related illness should be carefully monitored [105]. For patients traveling in areas without reliable access to a pharmacy, it may be prudent to carry a supply of vitamin K for reversing the effects of warfarin should the INR become excessive [104]. Time zone differences should be taken into account when planning communication between patients and anticoagulation providers [104]. The importance of proper hydration and avoiding prolonged immobility during long-haul air travel or car travel should also be emphasized [106].

### **Guidance Statements**

*When patients receiving warfarin therapy for VTE treatment travel outside of their healthcare system’s service area we suggest the following:*

- *Ensure that a sufficient supply of warfarin is available to cover the duration of travel*.
- *Make arrangements for ongoing INR monitoring.*
- *Ensure a plan for ongoing communication between the patient and anticoagulation provider is in place.*

## Conclusion

Expanding anticoagulant therapy options for treating VTE offer the potential for patients to receive more personalized therapeutic plans than have heretofore been possible. Well-managed warfarin therapy remains an important anticoagulant option and it is hoped that anticoagulation providers will find the guidance contained in this article increases their ability to achieve optimal outcomes for their patients with VTE (Table 8).

**Table 8:** Summary of guidance statements

Question	Guidance statement
(1) Who are good candidates for warfarin therapy versus the direct oral anticoagulants?	For patients with CrCl < 30 mL/min (estimated using the Cockroft–Gault equation) we suggest warfarin is the preferred anticoagulant. We also suggest vigilant monitoring including more frequent INR testing and bleeding risk assessment in patients with CrCl < 30 mL/min For patients with a history of poor medication adherence we suggest warfarin is the preferred oral anticoagulant. However, the requirement for routine INR monitoring of warfarin may be less than ideal for patients with restricted mobility, poor venous access, or other barriers to successful INR monitoring unless they are suitable candidates for self-testing at home using point-of-care INR monitoring devices (see below). In patients at high risk for bleeding complications; warfarin has the advantage of a proven antidote and DOACs have less major and/or clinically relevant non-major bleeding. These factors need to be incorporated into shared decision making with patients. When avoiding drugs known to interact with a given anticoagulant is not an option we suggest that warfarin is preferred because dose adjustments based on INR monitoring can facilitate titration of the anticoagulant response. We suggest that anticoagulation providers thoroughly discuss the advantages and disadvantages of available anticoagulants with patients and initiate therapy for VTE based on appropriate selection criteria and patient preference. For VTE treatment during pregnancy we suggest against using warfarin or DOACs. For VTE treatment in breastfeeding mothers we suggest that warfarin therapy is the best oral anticoagulant option. For patient with VTE associated with APLA syndrome, we suggest warfarin adjusted to a target INR range 2.0–3.0 is the best option for long-term treatment [43]
(2) How should warfarin be initiated?	During warfarin initiation for VTE treatment we suggest the following: Initiate warfarin as soon as possible following diagnosis of VTE, preferably on the same day, in combination with UFH, LMWH or fondaparinux The initial dose of warfarin should be 5 or 10 mg for most patients. Beginning on day three of therapy, INRs should be measured daily and warfarin doses adjusted to achieve an INR ≥ 2.0 as soon after day five of overlap therapy as possible. We suggest against using pharmacogenomic testing to determine initial warfarin doses for most patients.
(3) How can I optimize anticoagulation control?	When determining warfarin doses during VTE treatment we suggest using computer-aided warfarin dosing programs or validated dosing algorithms over an ad hoc approach We suggest enrolling patients with VTE in an AMS but when such services are not available, individual clinicians should strive to implement a similar structured care process. For treatment of VTE, we suggest PST and PSM should be accompanied by patient education and reserved for motivated patients who can demonstrate competency with self-testing equipment. For patients with previously stable therapeutic INRs presenting with a single out-of-range INR of ≤ 0.3 below or above the therapeutic range we suggest continuing the current warfarin dose and retesting the INR within 7 to 14 days. We suggest against the routine use of boost or skipped warfarin doses for unexplained slightly out of range INRs.
(4) How do I manage warfarin during invasive procedures?	For patients requiring invasive procedures during warfarin therapy for VTE we suggest the following: Careful coordination of periprocedural warfarin management involving the anticoagulation provider, person performing the procedure, and the patient Continuing warfarin during dental work, cataract surgery, minor dermatologic procedures and other procedures associated with minimal bleeding risk over interrupting warfarin therapy Stopping warfarin 4–5 days prior to the procedure to return the INR to near-normal values for procedures with high bleeding potential. No bridge therapy for most patients (exceptions might include VTE within the previous month, prior history of recurrent VTE during anticoagulation therapy interruption, undergoing a procedure with high inherent risk for VTE such as joint replacement surgery or major abdominal cancer resection)
(5) How do I manage warfarin-induced over-anticoagulation and/or bleeding	For non-bleeding patients presenting with an elevated INR we suggest the following: Withholding warfarin alone or in combination with 1.25 mg to 2.5 mg or oral vitamin K for INRs between 4.5 and 10.0. 2.5 mg of oral vitamin K for INRs > 10.0. For warfarin-related major bleeding we suggest rapid reversal of anticoagulation with 5 mg to 10 mg intravenous vitamin K and 4-factor non-activated PCC in conjunction with general supportive care and bleeding site interventions.
(6) How do I manage sub-therapeutic anticoagulation and recurrent VTE?	For most patients with VTE and subtherapeutic warfarin anticoagulation we suggest re-establishing therapeutic anticoagulation as quickly as possible without bridge therapy. For recurrent VTE not associated with subtherapeutic warfarin anticoagulation we suggest either increasing the target INR range or switching to an alternative anticoagulant.
(7) How do I manage warfarin drug–drug and drug-dietary interactions?	Following coadministration of drugs with the potential to interact with warfarin or significant changes in dietary vitamin K intake we suggest more frequent INR testing and warfarin dose titration as needed until INR stability is re-established. We suggest against routine use of daily low-dose vitamin K supplements as a means to improve therapeutic INR control or clinical outcomes during warfarin therapy.
(8) How do I switch between anticoagulants?	If a patient with VTE requires a switch from a DOAC to warfarin, we suggest one of the following approaches: Follow the information contained in the applicable product labeling For patients at low risk of VTE recurrence, we suggest avoiding LMWH bridge therapy For patients at high risk of VTE recurrence, we suggest overlapping apixaban, rivaroxaban, edoxaban, or dabigatran with warfarin therapy for at least three days combined with measuring the INR just before the next DOAC dose to limit any DOAC effect on the INR and discontinue DOAC therapy when an INR ≥ 2.0 is achieved.
(9) What is the appropriate follow-up and care transitions strategy?	For patients requiring warfarin dose adjustments for out of range INRs we suggest rechecking the INR within 7 days after INRs ≥ 4.0 or ≤ 1.5, and within 14 days following INRs 3.1 to 3.9 or 1.6 to 1.9 Following an INR > 5.0 we suggest rechecking the INR within 3 days. Following vitamin K administration for excessive anticoagulation we suggest rechecking the INR the next day When warfarin is being overlapped with parenteral anticoagulation therapy during initiation of acute VTE treatment we suggest that INRs be checked daily beginning on day three of therapy until the INR is ≥ 2.0 During the first three months of warfarin therapy for VTE we suggest that INR recall intervals not exceed 6 weeks For patients demonstrating consistently stable INRs after three months of warfarin therapy for VTE we suggest that INR recall intervals can be extended up to 12 weeks When patients receiving warfarin for VTE therapy transition between healthcare sites we suggest dedicated anticoagulation providers assume responsibility for care coordination using a structured approach We suggest that all patients and their caregivers receive patient-centered education regarding warfarin use for VTE treatment at the initiation of therapy and periodically thereafter
(10) How do I manage challenging clinical situations?	For patients with liver disease complicated by VTE we suggest against warfarin in patients with multiple or serious medical comorbidities and inability to closely monitor anticoagulation When warfarin therapy is deemed necessary in patients with liver disease we suggest a starting dose of 1 mg daily with careful dose titration to achieve an INR between 2 and 3. For patients with esophageal varices who require warfarin therapy we suggest banding of varices prior to beginning therapy when possible. To ensure patients on warfarin are not lost to follow up we suggest use of a systematic tracking process For patients who miss scheduled INR tests we suggest the use of non-threatening reminders delivered via phone, text message, email, or regular mail For patients having difficulty following warfarin dose instructions we suggest anticoagulation providers explore the use of pillboxes, calendars, diaries, electronic reminders, and written instructions as means to improve adherence When patients receiving warfarin therapy for VTE treatment travel outside of their healthcare system’s service area we suggest the following: Ensure that a sufficient supply of warfarin is available to cover the duration of travel Make arrangements for ongoing INR monitoring. Ensure a plan for ongoing communication between the patient and anticoagulation provider is in place
